# Early Adversity and Later Health: The Pathway of Social Relationships in Adulthood in HRS and ELSA

**DOI:** 10.1093/geroni/igab046.1863

**Published:** 2021-12-17

**Authors:** Andrew Steptoe, William Chopik, Amanda Leggett, Jooyoung Kong, Courtney Polenick, Yin Liu

**Affiliations:** 1 University College London, London, England, United Kingdom; 2 Michigan State University, East Lansing, Michigan, United States; 3 University of Michigan, Ypsilanti, Michigan, United States; 4 University of Wisconsin-Madison, Madison, Wisconsin, United States; 5 University of Michigan, Ann Arbor, Michigan, United States; 6 Utah State University, Logan, Utah, United States

## Abstract

Early adversity is associated with compromised health and well-being in later life, but whether social functioning mediate the association is unclear. We examined 2 longitudinal samples of older adults (>= 50 years) whose baseline surveys were between 2006 and 2008 with follow-up until 2016 in the Health and Retirement Study (HRS, n = 15,946) and its sister study in England (ELSA, n = 9,692). Health outcomes included depressive symptoms, chronic health conditions, and subjective memory complaints. Social relationships were measured by contacts, relationship strains, and feelings of loneliness. Early adversity was measured by parental physical abuse and alcohol and drug problems in the family before the age of 16. Patterns of association were similar in these 2 samples, where social contacts decreased over time, while relationship strains and loneliness increased especially for older adults with early trauma, which in turn mediated the associations between early adversity and poorer later health.

